# Supplementary material for: Novel Insights into Pigment Composition and Molecular Mechanisms Governing Flower Coloration in Rose Cultivars Exhibiting Diverse Petal Hues
Source: Plants (Basel). 2024 Nov 29;13(23):3353. doi: 10.3390/plants13233353 (PMC11644816; doi:10.3390/plants13233353)
Supplement: Supplementary file 1 [file plants-13-03353-s001.zip › plants-3283618-supplementary.pdf]

Supplementary data

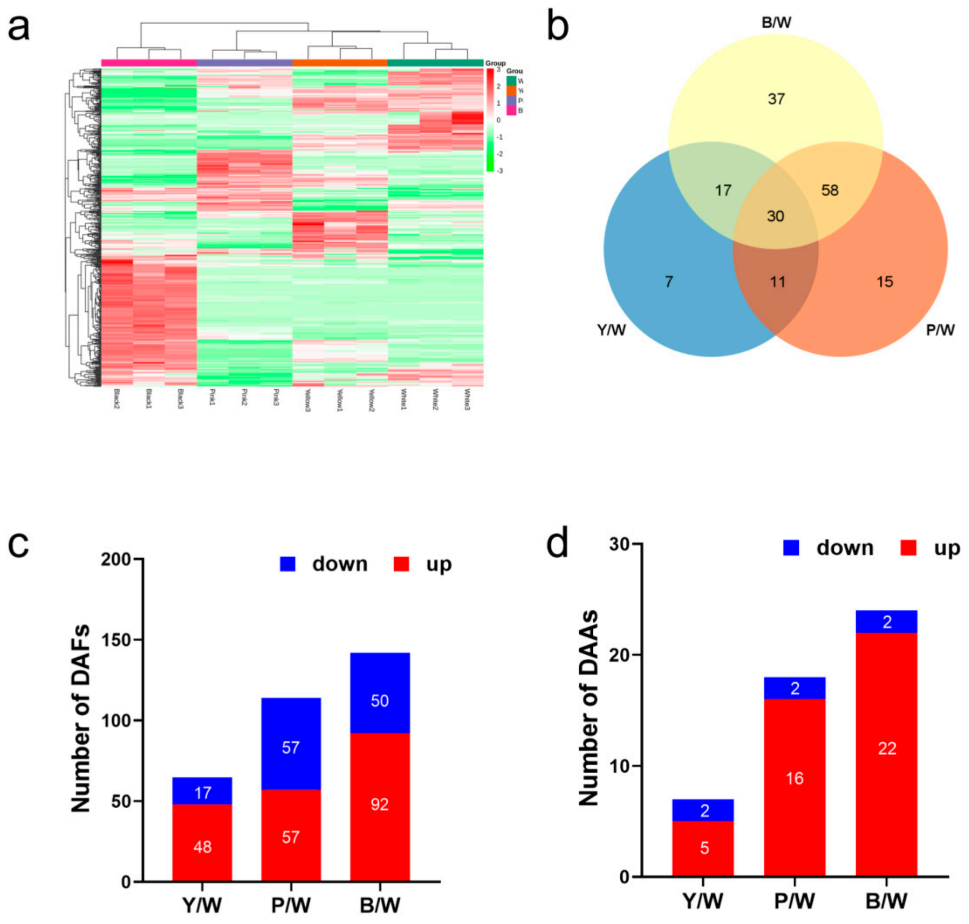

Supplementary Figure S1 Differentially accumulated metabolites analysis of metabolome. a Hierarchical cluster analysis of differential metabolites of different colored petals. **b-c** Differential accumulation of flavonoid amounts in Y/W, P/W and B/W. **d** Differential accumulation of anthocyanin amounts in Y/W, P/W, and B/W.

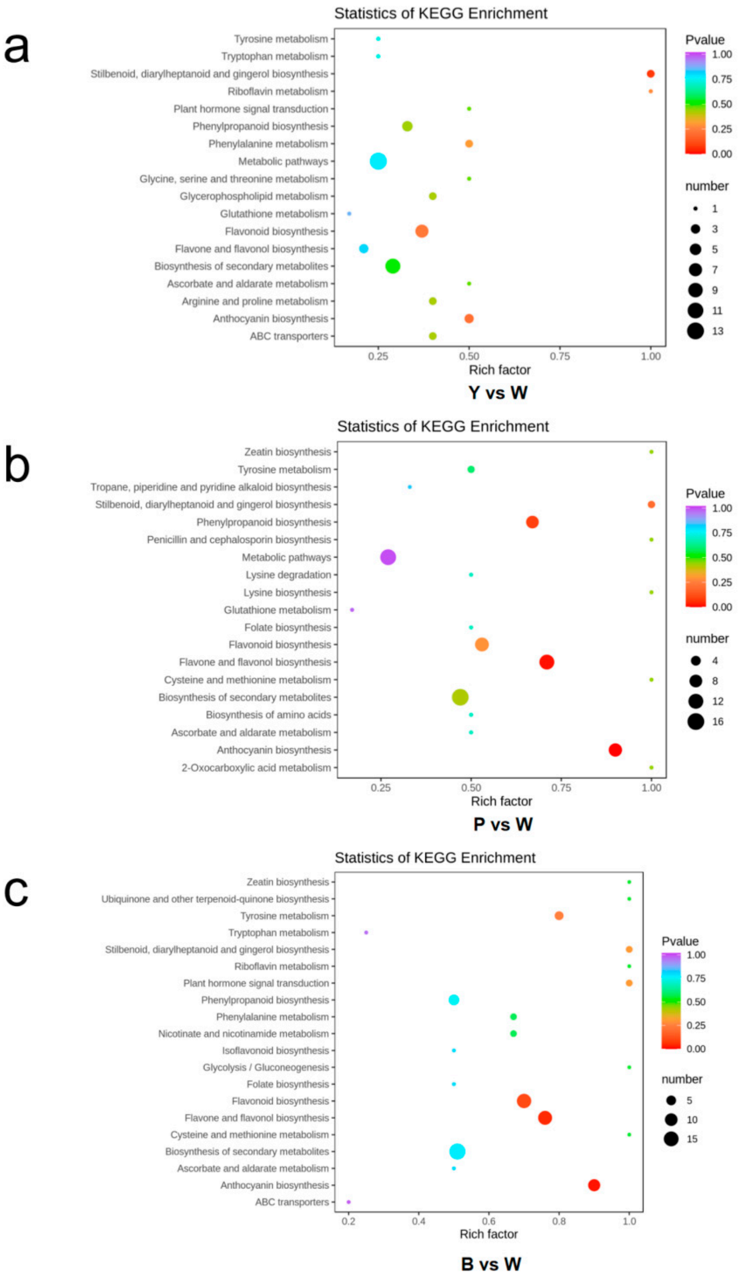

Supplementary Figure S2 KEGG enrichment map of differential metabolites. a Y vs W, b R vs W, c B vs W.

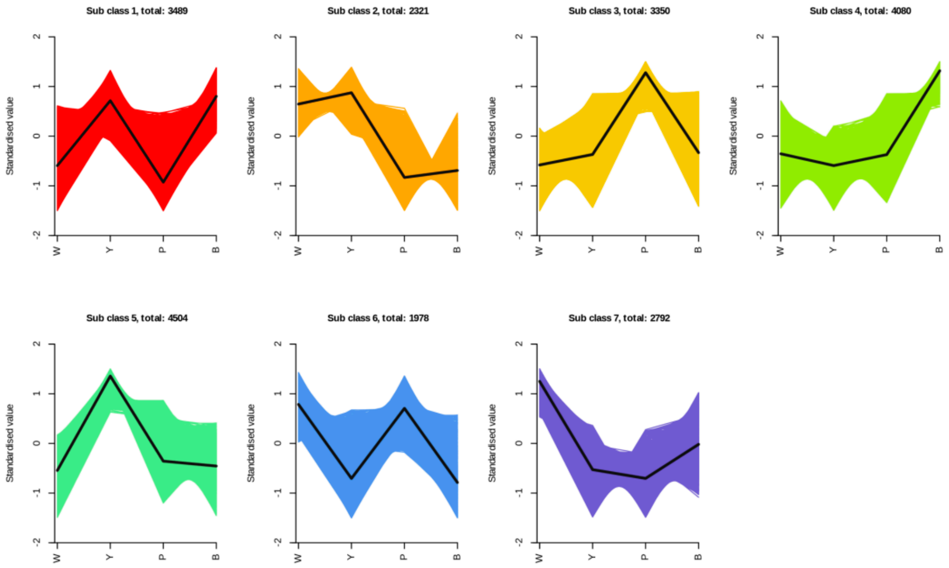

Supplementary Figure S3 Differential gene Kmeans map.

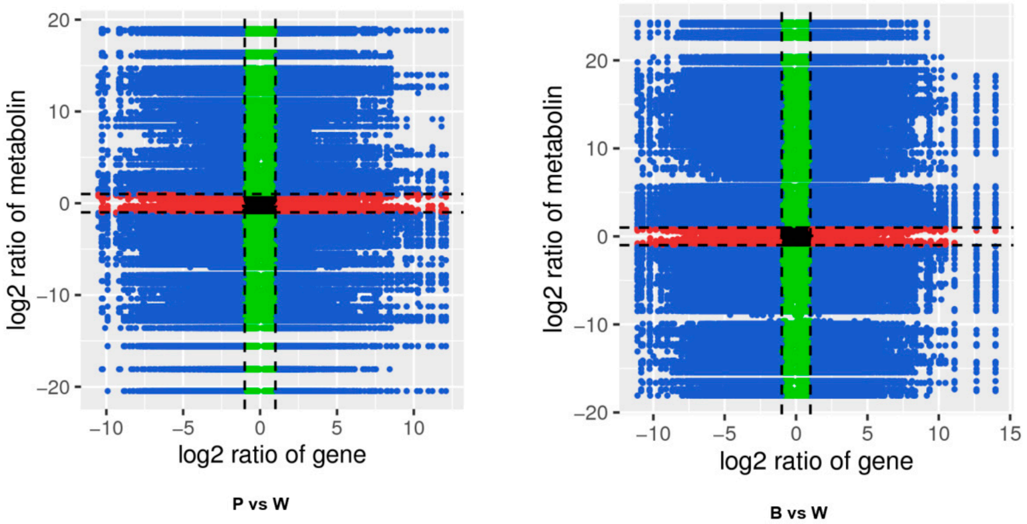

Supplementary Figure S4 Correlation analysis nine quadrants.

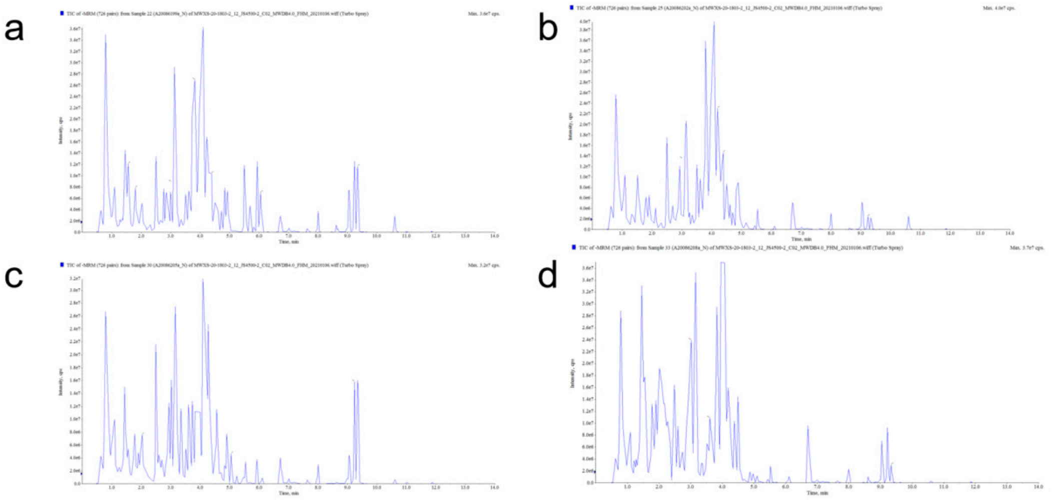

Supplementary Figure S5 Total ion flow diagram for mass spectrometric analysis of mixed metabolite samples. a-d represent white samples, yellow samples, pink samples and black-red samples respectively.

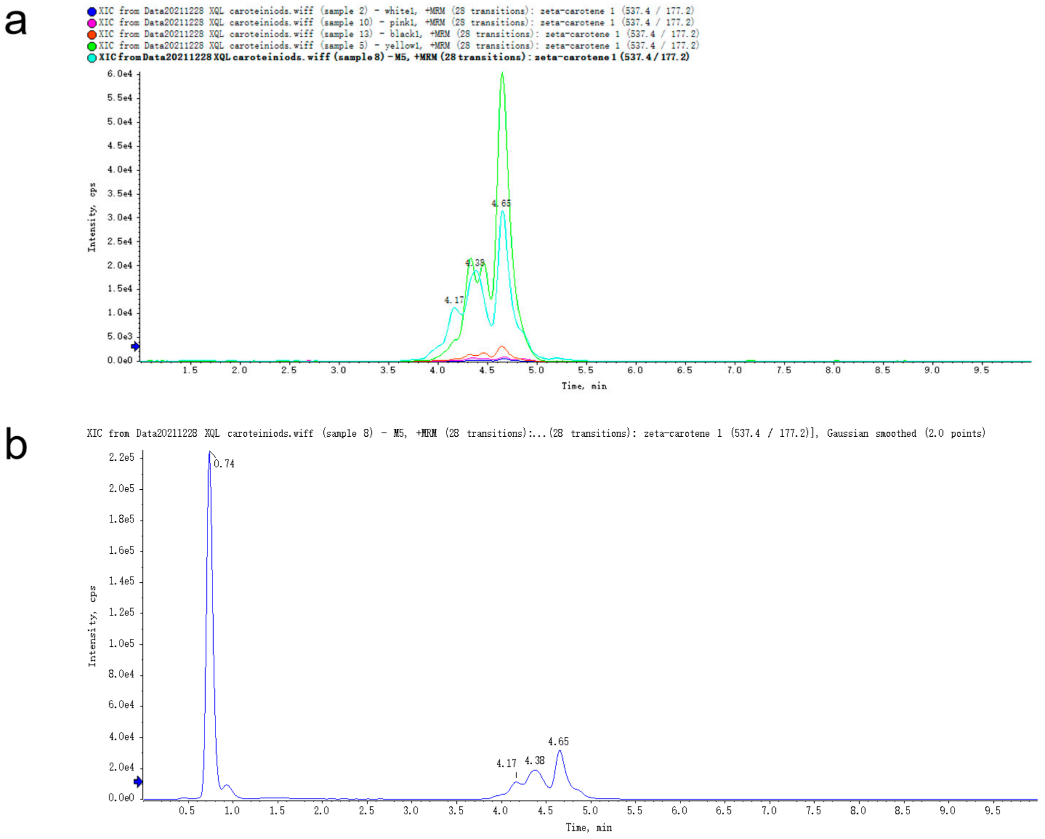

Supplementary Figure S6 HPCL peak. a Plot of standard peaks. **b** Sample peak graph.

Supplementary Table S1 Primers for PCR amplification and quantification. All the primers we

used were designed by Primer premier 5.0 software.

Primer names	Sequences (5' →3')
q*RhCHS*-F	GACAGCAACTCCTCCCAATTGT
q*RhCHS*-R	TTGTCTTGCATCAAGTGAAGGG
q*RhCHI*-F	TGGAAGACCAAGAAAGGAACTGA
q*RhCHI*-R	GCCAACCTATCCCTCACTGAAC
q*RhDFR*-F	TGGTGTCTTCCACTCTGCTTCT
q*RhDFR*-R	GTGAGAACCACACGCCTTAGAG
q*RhANS*-F	GGAGGACTATTTTTTCCACTGTGT
q*RhANS*-R	AATCCCAAGCCAAGTGAGAGTA
q*RhPSY*-F	GTCGCAAAGTACCCAGTGGATA
q*RhPSY*-R	ATCAGTCCAACAGTTCCAGCAA
q*RhZDS*-F	TTTGGCTTTATTCCCATCATC
q*RhZDS*-R	ATTAGCGGCTTTTGTATTCCC
q*RhLYCB*-F	TTTGATGTGGATAAGATGGTTTTC
q*RhLYCB*-R	ACTCTTTGAGGGAGCACCG
q*RhCHYB*-F	CAACGAATCCTTGAGGCTTTAC
q*RhCHYB*-R	CTGCGTCATCCCACAGTGTT
q*RhZEP*-F	TGTCTCTCTAAAATCCGCCAAAG
q*RhZEP*-R	TTCCTGAAAAATCAAGAACCCC
q*RhPDS*-F	GGAGACTGGATTACAACCCGAC
q*RhPDS*-R	GAATGTGTGGTTCGTCCTCCTC
q*RhMYB1*-F	CGAAGGATGGACTCAACAAAG
q*RhMYB1*-R	ATCATCTGAAATGCTGCCTCTC
q*RhMYB113*-F	TTCGGTGGCTAAACTACCTTCG
q*RhMYB113*-R	CACATCATTACCAGTCCTTCCC
q*RhMYB1R1*-F	AGGAAGAGCGTCAGTCTCAAC
q*RhMYB1R1*-R	TCTCCAATCTCCTTTCCCTAC
q*RhUBI6*-F	GGAGTCGGCCAGGTGCGTTT
q*RhUBI*6-R	TGGGCTGGGCATTCTTCACCAC

Supplementary Table S2 Differential accumulation of metabolites in the Y/W, P/W and B/W(.xlsx)

Supplementary Table S3 Differential accumulation of flavonoids in Y/W, P/W and B/W and analysis of transcription factors in W, Y, P and B samples(.xlsx)

Supplementary Table S4 The raw data of transcriptome between four rose cultivars.

Sample	Raw Reads	Clean Reads	Clean Base(G)	Q30(%)
**White1**	48376152	45104544	6.77	89.35
**White2**	61194614	54648266	8.2	89.8
**White3**	66790254	58497306	8.77	89.88
**Yellow1**	58421930	52333474	7.85	89.37
**Yellow2**	54997090	49386512	7.41	89.84
**Yellow3**	60991966	56764394	8.51	89.41
**Pink1**	57350242	51738468	7.7	89.42
**Pink2**	69923994	63905168	9.59	89.02
**Pink3**	57210284	51987148	7.8	89.38
**Black1**	49887466	45849394	6.88	89.2
**Black2**	47830736	44948310	6.74	88.93
**Black3**	52197306	48325326	7.25	88.93

Supplementary Table S5 Associations between metabolites and transcriptomic analysis(.xlsx)
